# Ineffectiveness of Transfixing Sutures in Enhancing Weight Loss Following Gastric Plication: A Case Report

**DOI:** 10.7759/cureus.73770

**Published:** 2024-11-15

**Authors:** Juan Carlos Del Castillo, Alvaro Velasquez, Valeria Agredo Garcia, David Alexander Vernaza Trujillo

**Affiliations:** 1 Bariatric Surgery, Clínica Nuestra Señora de Los Remedios, Cali, COL; 2 Gastrointestinal Surgery, Clínica Nuestra Señora de Los Remedios, Cali, COL; 3 Epidemiology, Fundación Universitaria del Área Andina, Bogotá, COL; 4 Interinstitutional Group of Internal Medicine 1 (GIMI1), Universidad Libre, Cali, COL

**Keywords:** endoscopic gastric plication, gastric bypass surgery, gastric sleeve surgery, laparoscopic gastric plication, obesity treatment, weight loss and obesity

## Abstract

Endoscopic gastric plication is an emerging procedure for obesity management, facilitated by advances in intraluminal suturing technology. However, concerns persist regarding the complete transfixion of sutures and their long-term durability. We present the case of a 28-year-old patient with a BMI of 31 kg/m² who underwent laparoscopic gastric plication using nonabsorbable sutures to ensure complete transfixion under direct visualization. The procedure’s effectiveness and durability were evaluated through clinical and endoscopic follow-up.

## Introduction

Obesity is a global health challenge that requires effective and sustainable solutions. Endoscopic gastric plication (EGP) has demonstrated promising weight loss outcomes, typically resulting in a loss of 9-13.6 kg over six to 12 months. However, concerns persist regarding the long-term efficacy and integrity of sutures. This report explores laparoscopic gastric plication (LGP) as an alternative that enables complete suture transfixion under direct visualization, potentially enhancing the durability and effectiveness of the procedure [1].

EGP is an emerging procedure in obesity management, supported by technological advances that allow for intraluminal suturing. According to recent guidelines, endoscopic bariatric and metabolic therapies, including gastric plication, are recommended for patients with a BMI of 30 kg/m² or higher, regardless of comorbidities, and for patients with a BMI of 27-29.9 kg/m² who have at least one obesity-related comorbidity [2]. Furthermore, these procedures provide a treatment option for individuals who may not qualify for or prefer to avoid traditional bariatric surgery, thus filling a significant treatment gap [3].

We present the case of a 28-year-old patient with a BMI of 31 kg/m² who sought LGP following unsuccessful weight loss attempts with diet and cosmetic interventions. This case report evaluates the effectiveness and durability of the procedure, utilizing nonabsorbable sutures to achieve full-thickness transfixion under direct visualization, with clinical and endoscopic follow-up to assess outcomes.

The present article was prepared in accordance with the CARE guidelines for case reports.

## Case presentation

A 28-year-old female patient sought weight loss after multiple unsuccessful attempts with diets and previous cosmetic surgeries. She had a BMI of 31 kg/m² and no significant comorbidities. Her goal was to lose approximately 11.3 kg (25 pounds).

During the initial evaluation, various therapeutic options were discussed, including EGP. However, due to financial limitations, the patient was unable to pursue this approach because of its high cost. As an alternative, LGP was proposed, and the patient accepted after a detailed explanation of the procedure and its implications.

Before surgery, the patient was evaluated by a multidisciplinary team, including nutrition, psychology, and anesthesiology. Informed consents were obtained, and all ethical and legal requirements established by the medical institution were met.

The patient underwent LGP under general anesthesia. The surgical approach involved the placement of three laparoscopic ports: one 12 mm port and two 5 mm ports, along with the use of a handcrafted internal liver retractor to optimize exposure of the surgical field. Pneumoperitoneum was established using CO₂ insufflation, maintaining an intraperitoneal pressure of 12 mmHg and a flow rate of 15 L/min. Visualization of the operative field was achieved with a 5 mm laparoscopic camera, and Storz instruments were used throughout the procedure.

**Figure 1:**
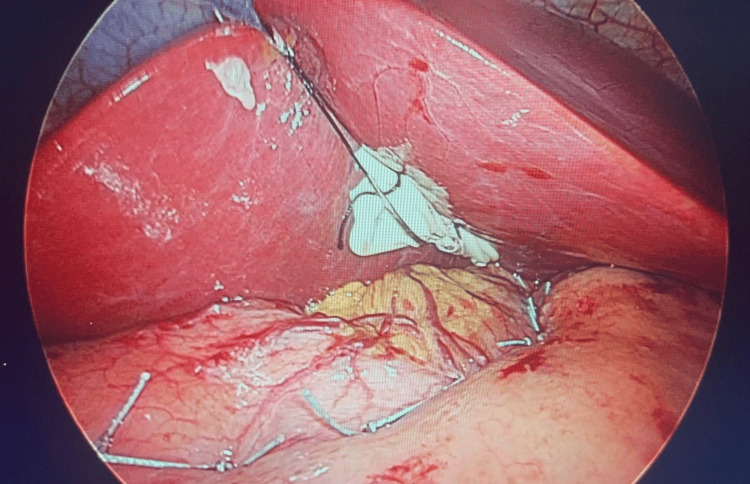
Placement of sutures along the stomach using a 40 French bougie as a guide

**Figure 2:**
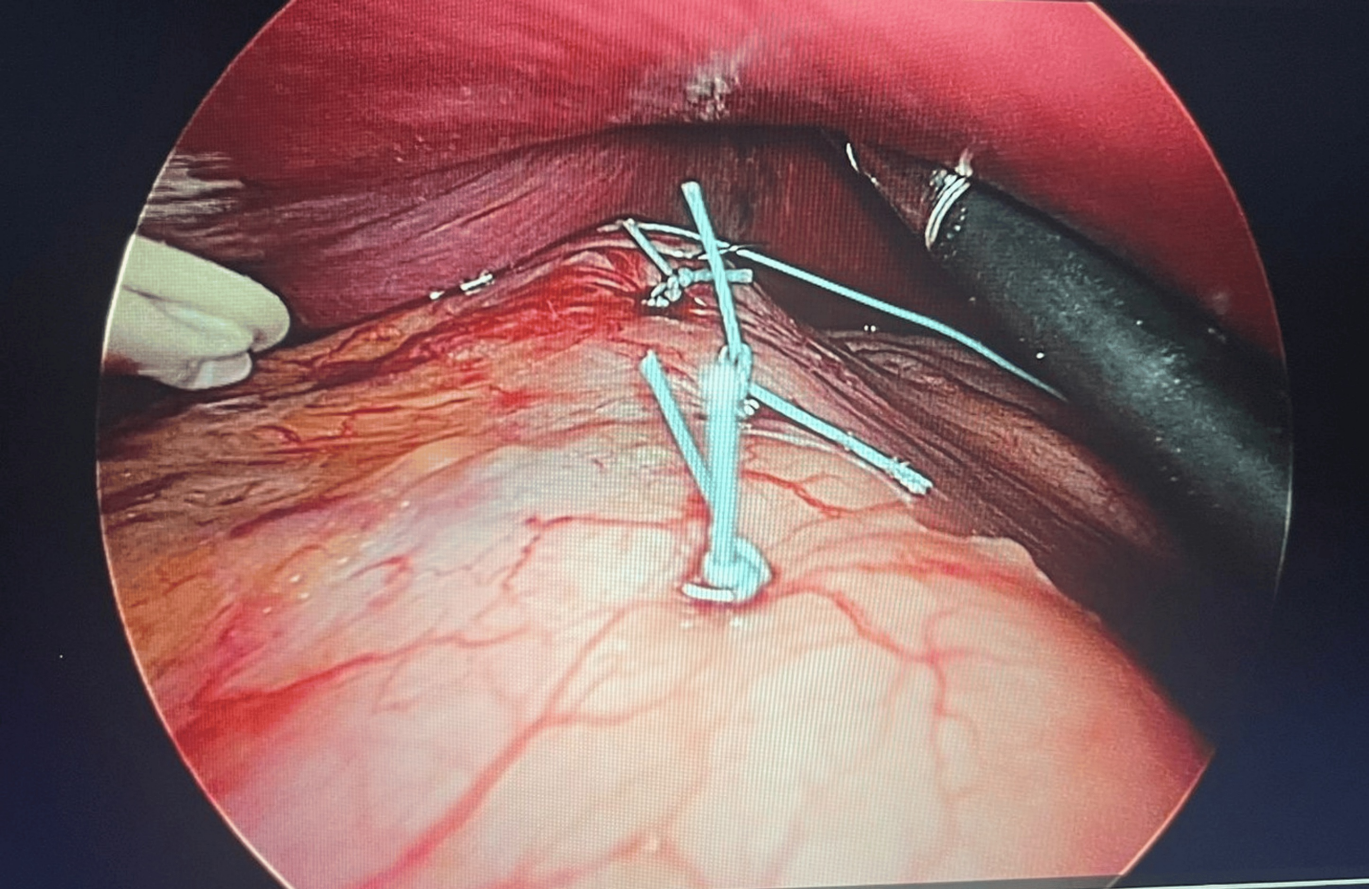
Placement of multiple separated full-thickness transfixing sutures along the stomach using a 40 French bougie as a guide

A 40-French bougie was introduced to serve as a guide for the gastric lumen, ensuring consistent dimensions. Subsequently, 12 transfixing sutures using 2-0 nonabsorbable Ethibond were applied from the His angle to the gastric antrum, with the intention of creating a sleeve-like effect by folding the stomach and reducing its capacity (Figure 1). The sutures were carefully placed to achieve full-thickness apposition, ensuring the durability of the fold. The entire procedure was completed within 35 minutes (Figure 2).

To verify the integrity of the newly created gastric fold, a methylene blue leak test was performed intraoperatively. No leaks were observed, indicating successful closure and secure apposition of the sutures.

The closure of the surgical incisions involved suturing the 12 mm umbilical port site with 1-0 Vicryl, and the skin was closed with 4-0 Monocryl.

Postoperatively, the patient had an uneventful recovery, with stable vital signs and no immediate complications. She was monitored overnight in the hospital and discharged the following day after meeting all discharge criteria, including tolerance of oral intake and stable ambulation. Multidisciplinary follow-up appointments were arranged to ensure optimal recovery and support. A nutritional consultation was scheduled for day 5 postoperatively to initiate dietary adjustments, and medical and psychological follow-ups were planned for day 8 to address any concerns and support the patient's adaptation to lifestyle changes following the procedure.

Thirty days after surgery, the patient started a solid diet and reported no sensation of restriction when eating. Given this observation, and to evaluate the procedure's effectiveness, an upper gastrointestinal endoscopy was performed.

**Figure 3:**
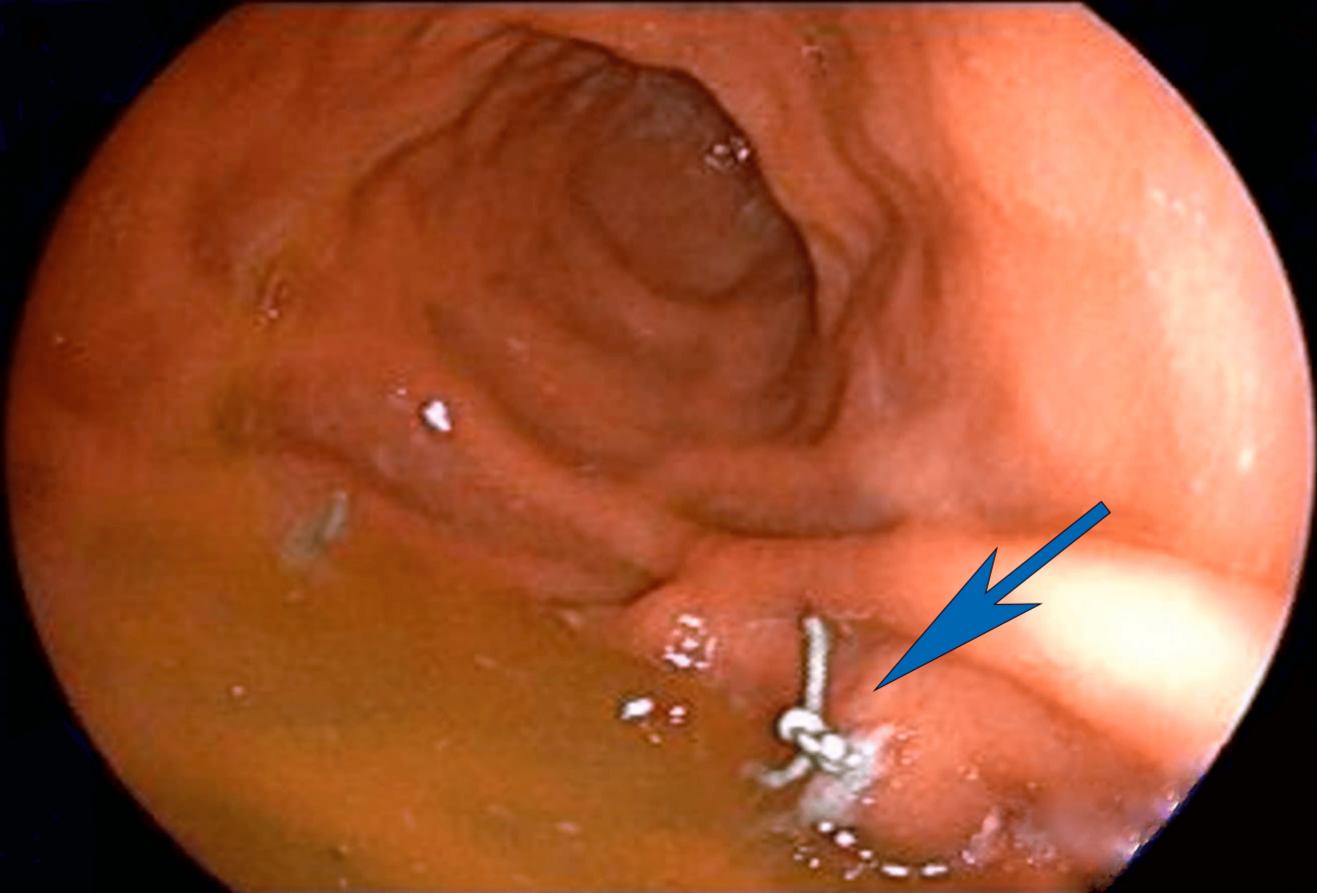
Endoscopy at six weeks postoperative showing intragastric knots. All sutures have penetrated into the gastric cavity, resulting in complete loss of the plication in 100% of the transfixing sutures

The endoscopy revealed a stomach with a normal appearance and 100% expansion. Suture knots were observed on the posterior gastric wall, but no suture material was evident collapsing the stomach. All knots were present within the gastric lumen, indicating that they had penetrated the gastric cavity and lost their restrictive effect on all of the sutures (Figure 3).

## Discussion

LGP and EGP, including techniques like the Apollo Method, are restrictive procedures aimed at reducing gastric volume without tissue resection, offering a less invasive alternative to procedures such as sleeve gastrectomy and gastric bypass. In this clinical case, the patient initially showed favorable weight loss outcomes post-procedure but experienced a rapid loss of restrictive effect within one month, consistent with reports of relatively high failure rates for these restrictive techniques [1].

The Apollo Method, an endoscopic approach, utilizes a transmural suturing system to fold the stomach internally without requiring external incisions. This technique, performed with an endoscopic suturing device, typically involves six to eight plications, compressing the gastric cavity and creating a restrictive effect akin to sleeve gastrectomy [4]. Early studies suggest promising outcomes, with a reported weight loss of 55.3% of excess weight at six months post-procedure [4]. However, concerns regarding the long-term durability of the sutures persist, with incomplete transfixion contributing to the loss of the restrictive effect, as observed in this case [5].

LGP, in contrast, offers better visualization and control of sutures, allowing for complete transfixion of the gastric wall under direct vision. The procedure typically involves placing approximately 12 nonabsorbable sutures from the His angle to the gastric antrum. Although LGP is technically safe and less invasive than other bariatric surgeries, challenges regarding suture durability remain, as seen in this case, where endoscopic follow-up revealed that the knots had penetrated the gastric cavity, leading to the loss of the restrictive effect [6,7].

Studies, such as those by Ji et al., highlight that LGP has a relatively high revision rate. They found that, although LGP is less invasive and avoids tissue resection, up to 15% of patients may experience insufficient weight loss or complications that necessitate revision [8]. In this case, the early loss of the restrictive effect may make the patient a candidate for surgical revision or conversion to a more invasive procedure, as suggested by previous studies.

The revision rate for LGP is a critical factor. Zerrweck et al. conducted a retrospective study involving 100 LGP patients and found that 30% required re-intervention due to ineffectiveness or severe complications, such as epigastric pain and gastroesophageal reflux [9]. This finding is particularly relevant to this case, where the loss of gastric restriction occurred during early follow-up. In such cases, more invasive conversion procedures, such as gastric bypass or sleeve gastrectomy, may be considered, as these procedures have proven to be safe and effective in addressing LGP failure [10,11].

Some studies have shown that converting LGP to more invasive procedures may be necessary for achieving long-term weight loss outcomes. For instance, Nevo et al. described the conversion of a failed LGP to a one-anastomosis gastric bypass, resulting in sustained weight loss [12]. While these conversion procedures can offer better long-term results, they come with increased surgical risks and higher costs, which could be a limiting factor for some patients [13].

Although LGP is less expensive than other bariatric surgeries, such as gastric bypass or sleeve gastrectomy [14,15], the initial cost savings may be offset by the need for revisions or conversions in cases of procedure failure. In this case, the patient opted for LGP due to financial constraints, but the possibility of re-intervention could lead to increased long-term costs. Zerrweck et al. also found that conversion to more invasive surgeries, like gastric bypass or sleeve gastrectomy, significantly improved weight loss outcomes but may also involve longer hospital stays and higher complication rates [9].

## Conclusions

This case underscores the limitations of LGP as an alternative to EGP in obesity management. While LGP offers the advantage of being minimally invasive, allowing for better visualization and complete suture transfixion, its long-term effectiveness is compromised, as demonstrated by the early loss of restrictive effect in this patient. Both LGP and EGP face challenges related to suture durability, often necessitating re-interventions or conversion to more invasive procedures such as gastric bypass or sleeve gastrectomy, which have demonstrated sustained efficacy in achieving long-term weight loss.

While LGP remains a more cost-effective option, the initial savings may be outweighed by the potential need for surgical revisions. This is a key consideration in clinical decision-making, particularly for patients with financial constraints. Continued exploration of surgical technique advancements and improved suture materials is essential to enhance the durability and long-term success of these procedures. Furthermore, careful evaluation of patient expectations and available long-term treatment options for obesity is critical in guiding effective management strategies.
